# Clinical impact of customised positive airway pressure (PAP) therapy interfaces versus usual care in the treatment of patients with sleep-disordered breathing (3DPiPPIn): a randomised controlled trial protocol

**DOI:** 10.1136/bmjopen-2024-087234

**Published:** 2024-11-14

**Authors:** Stephanie K Mansell, Swapna Mandal, Deborah Ridout, Oliver Olsen, Francesca Gowing, Cherry Kilbride, Stephen T Hilton, Eleanor Main, Silvia Schievano

**Affiliations:** 1 University College London, London, UK; 2 Royal Free London NHS Foundation Trust, London, UK; 3 Oliver Olsen, Guildford, UK

**Keywords:** Sleep medicine, Randomized Controlled Trial, Health informatics

## Abstract

**Introduction:**

Sleep-disordered breathing affects 1.6 million people in the UK. The recognised treatment is positive airway pressure (PAP) therapy, delivered via a generic conventional interface (mask). PAP therapy improves morbidity, mortality and quality of life, but treatment effectiveness depends on interface fit and tolerance. Interface side effects include pressure ulcers, skin reactions and interface leak. Three-dimensional (3D) printing is an innovative technology that can produce customised interfaces.

**Aims:**

The primary aim is to assess the impact of customised versus conventional interfaces on residual Apnoea Hypopnea Index at 6 months.

**Methods and analysis:**

This is a randomised control trial via block randomisation, minimised by age >65 and ethnicity, using a computerised random number generator. Patients with sleep-disordered breathing under the care of the Royal Free London NHS Foundation Trust will be recruited. Patients new to therapy will be randomised to customised interface or conventional interface for 6 months. A sample size of 160 is required for 80% power with a significance of 5%, accounting for a 20% dropout rate. Descriptive statistics will report demographics. The primary and secondary outcomes will be compared using linear regression adjusted for baseline score.

**Ethics and dissemination:**

This protocol has been approved by the Hampshire B Research Ethics Committee (REC reference: 22/SC/0405). Results will be disseminated to healthcare professionals and patients through conferences, open-access journals, newsletters, a study webpage, infographics, animations, social media and healthcare awards.

**ISRCTN registration number:**

74082423.

**STRENGTHS AND LIMITATIONS OF THIS STUDY**

- The randomised control trial design enhances the reliability of findings by reducing selection bias.
- Block randomisation minimised by age and ethnicity ensures a balanced representation of these variables, which are most likely to affect facial geometries across treatment groups.
- The well-defined target population ensures that results will apply to patients with sleep-disordered breathing.
- The lack of blinding and the single-site design could introduce bias in assessing outcomes.
- It has not been possible to include a health economics assessment due to the constraints of funding.

## Introduction

 Sleep-disordered breathing (SDB) is a term encompassing conditions including obstructive sleep apnoea (OSA), complex sleep apnoea and chronic ventilatory failure. Chronic ventilatory failure is commonly caused by chronic obstructive pulmonary disease, obesity-related respiratory failure, neuromuscular disease and chest wall deformity. Symptoms of SDB are multiple and include excessive daytime sleepiness, headaches, impaired cognition, breathlessness, sleep disturbance, nocturia, fatigue, memory loss and reduced libido.^1^ Untreated SDB increases the risk of cardiovascular events and is associated with more frequent healthcare utilisation, including hospital admissions.^1^ Additionally, untreated SDB influences societal factors, such as quality of life (QoL), road traffic accidents and productivity.^1^ A measure of severity in SDB is the Apnoea Hypopnea Index (AHI). Both continuous positive airway pressure (CPAP) and non-invasive ventilation (NIV), collectively referred to as positive airway pressure (PAP), can treat SDB and are crucial to improving patients’ QoL and health-related outcomes. PAP therapy is administered via a tight-fitting mask (interface) attached to the patient’s face. Outcomes such as residual AHI are critical clinical measures of PAP therapy effectiveness for individual patients.

The effectiveness of PAP therapy depends on how well the interface fits the user. Interface leak has been found to cause ineffective ventilation, high residual AHI, persistent nocturnal desaturations and treatment failure.^2^,^4^ Pressure ulcers are also a documented side effect of PAP therapy, which can impact the ability to concord with treatment.^5^

Interfaces are currently limited to conventional interfaces supplied by PAP device manufacturers, which come in limited sizes (small, medium large) and do not consider different facial geometries. Thus, ineffective therapy due to a lack of interface fit is commonly observed. Impaired effectiveness or poor treatment tolerance can reduce QoL, potentially affecting morbidity and mortality and escalating healthcare utilisation.

Three-dimensional (3D) printing is a pioneering technology, which has been used in a range of clinical environments. Several investigators have considered using 3D printing in developing customised interfaces. Case studies^6^,^11^ in paediatric patients demonstrated a significant reduction in interface leak with improved compliance, effectiveness of PAP therapy (reduced residual AHI) and health-related QoL. Hsu *et al*^12^ undertook a feasibility study with 40 participants comparing conventional and customised interfaces. They reported less headgear force was required in the customised group, which resulted in better comfort scores. A small (n=6) crossover study^13^ evaluated the feasibility of 3D-printed customised nasal interfaces and found interface leak comparable between customised and conventional interfaces. Additionally, they reported that the customised interfaces could deliver the required PAP therapy pressures. Similarly, Cheng *et al*^14^ assessed a customised nasal interface developed using 3D printing technology against a conventional cushion and reported a lower residual AHI in the customised interface. However, these studies are limited to a single ethnicity group, in those with OSA alone or very specific patient groups (paediatrics with facial deformities), meaning the results are not generalisable. Furthermore, research to date has only considered short-term outcomes.

This project aims to develop and assess the clinical utility of 3D printing to customise PAP therapy interfaces.

### Research question

What is the clinical impact of customised PAP therapy interfaces versus conventional interfaces in the treatment of patients with SDB?

### Hypothesis

This study hypothesises that customised interfaces will result in greater improvements in residual AHI compared with conventional interfaces, with better PAP concordance due to reduced discomfort and side effects.

### Primary research objective

A randomised controlled trial (RCT) to assess medium-term clinical effectiveness of customised interfaces versus conventional interfaces as assessed by the primary outcome of residual AHI (worse of two nights measured by cardiorespiratory sleep study) at the primary endpoint of 6 months.

### Secondary research objectives

To assess the clinical impact of customised interfaces on interface fit (leak), concordance with PAP therapy and symptoms compared with conventional interfaces.

## Methods and analysis

### Patient and public involvement

Service users were actively involved in the research design; specifically, they provided feedback on the proposed methodologies and dissemination plan and coproduced the lay summary. They reviewed and completed all the intended outcome measures. Participants provided feedback and ideas on reducing the burden of participating in research and codesigned the recruitment strategy. A patient/public advisory group will meet quarterly to provide input into the conduct of the trial.

### Study design

RCT, with patients randomised by block randomisation, minimised by age (<65, ≥65) and ethnicity (Caucasian, Asian and Black), to customised interface or conventional interface, by a computerised random number generator. The primary outcome is residual AHI at the primary end point of 6 months. Based on clinical expertise and published research, ethnicity and age are the biggest factors affecting facial geometry^15 16^ and thus have been included in the stratification.

### Patient entry

Patients will be recruited from those under the care of the sleep and ventilation service at the Royal Free London NHS Foundation Trust (RFL), United Kingdom (UK). The RCT has been designed as a pragmatic trial and is embedded into the existing clinical pathways.

### Inclusion criteria

- Diagnosis of sleep discorded breathing: AHI≥15.
- Patients naive to domiciliary PAP therapy.
- Age ≥18 years.

### Exclusion criteria

- AHI <15.
- Excessive facial hair which they are unwilling to shave.
- Age <18 years.
- Existing facial pressure ulcers.
- Unable to provide informed consent.
- Known allergy to silicone.
- Keloid scarring.
- Previous domiciliary PAP therapy.

### Withdrawal and stopping criteria

Participants will have the right to withdraw from the study at any time without providing a reason. Any data already collected will remain in the study.

### Data management

#### Sample size

A sample size of 160, with 80 per group, is required for a power of 80%, with a significance of 5%, assuming an effect size of 0.50 and allowing for a 20% dropout rate. The effect size was determined using data from previously published research^14^ compared with a cohort of randomly selected patients under the care of RFL. This method ensured that the power calculation was relevant to a United Kingdom (UK) population.

#### Analysis

Descriptive statistics will be used to report patient demographics. The hypothesis will be tested by comparing the difference between the groups in residual AHI (the primary outcome) at the primary endpoint (6 months) using linear regression adjusting for score at baseline and stratification variables (age as a binary variable and ethnicity) on an intention-to-treat basis. Residual AHI has been chosen as the primary outcome measure as, where interface fit is poor, residual AHI has been shown to be high. Furthermore, it is a measure of treatment effectiveness, which can be used for both CPAP and NIV. The pros and cons of each potential primary outcome measure were discussed with PPI workshop attendees who agreed AHI was a suitable and acceptable primary outcome. AHI will be measured for two nights via a home-based cardiorespiratory sleep study, and the worst (higher AHI) of the two nights will be used. The analyses will be repeated using the 3-month results as a secondary outcome measure.

For secondary outcome measures, differences between groups will be compared using linear regression adjusting for respective scores at baseline and stratification variables. The analyses will be repeated using the results at 3 months.

The interface questionnaire will be analysed using descriptive statistics, bivariate analysis and ordinal regression. Dropout rates between groups will be compared using a χ^2^ test. The analyses will be repeated using the results at 3 months.

#### Statistical software

Data will be collected in a RedCap database. Data will be analysed using Excel, SPSS and R.

### Trial design

#### Screening and recruitment

The principal investigator will screen potential participants from patients referred to the RFL sleep and ventilation service. Those whose initial sleep study results in an AHI≥15 will be considered for screening into the study. Screening data will be collected using the SEAR (screened, eligible, approached, randomised) framework.^17^ Demographic information on patients screened for the trial will include age, gender, ethnicity, screening ID and initials. Inclusion and exclusion criteria information will be collected using the electronic case report form (CRF) (collected via the RedCap Database), which allows the identification of ineligible participants and reasons for ineligibility. The date the trial was discussed and written information was provided to potential participants, as well as reasons for non-approach, will be recorded in the electronic CRF (collected by the RedCap database). Where participants decline to participate in the trial, their reason for refusal and subsequent treatment received will be collected using the electronic CRF (collected via the RedCap Database).

Patients will be identified as potential candidates at several points in the pathway depending on their diagnosis and the clinical pathway. Many patients will have completed a home-based cardiorespiratory sleep study, where patients have already provided permission to contact and are on the academic respiratory medicine permission to contact database, they will be sent the invitation letter and participants’ information sheet via their preferred method of contact. A follow-up phone call will be scheduled with a member of the research team. Where the patient has not already given permission to contact, a clinical appointment will be scheduled to explain their sleep study results and offer them the opportunity to participate in the trial.

The trial will be promoted via a trial website and social media. Posters will be displayed in appropriate clinical areas.

#### Baseline assessments

The following demographic and baseline data will be reported:

- Age.
- Ethnicity.
- Sex.
- Primary sleep-disordered breathing diagnosis.
- Pre-existing relevant medical history.
- Pre-existing relevant medication.
- Smoking status.
- Alcohol consumption.
- Baseline Epworth Sleepiness Score (ESS).^18^
- Sleepiness-Wakefulness Inability and Fatigue Test (SWIFT) questionnaire.^19^
- S3 Non-Invasive Ventilation (S3NIV).^20^
- Baseline AHI.
- Baseline body mass index.
- Baseline respiratory rate.
- Baseline SpO_2_^.^
- Baseline Waterlow score.
- Rockwood Frailty score.

#### Assessment

Measurement of the primary outcome at the primary endpoint—residual AHI at 6 months, will be assessed through a two-night home-based cardiorespiratory sleep study. The minimally clinically important difference for AHI has been proposed as five events/hour,^1^ but this has not been assessed in trials or clinical practice. A residual AHI of ≤7 events/hour has been found to indicate good control of SDB.^21 22^ AHI has been found to have some night-to-night variability in patients with mild and severe OSA,^23^ but this has only been investigated during diagnostics and not in response to PAP therapy. To navigate this issue, patients will undertake a home-based limited cardiorespiratory sleep study at baseline, 3 and 6 months to reassess AHI, with the worst (higher AHI) of the two nights being recorded.

Collecting the following measurements will facilitate the assessment of secondary outcome measures:

- Interface leak, measured as average L/min and percentage of intentional leak over 7 days from PAP therapy data downloads at 3 months and 6 months.
- Residual AHI will be measured using PAP therapy data downloads for further validation of AHI, while others have investigated the reliability of PAP therapy data downloads,^21 22^ the software and devices used in this study have not been tested.
- Incidence of pressure ulcers measured by the European Pressure Ulcer Advisory Panel score (0 to 4).^24^
- Compliance with PAP therapy, measured as hours and percentage of days PAP therapy used >4 hours/night over 28 days, measured at 3 months and 6 months in concordance with internationally recognised definitions of compliance.
- Symptoms measured by the ESS and SWIFT measured at baseline, 3 months and 6 months. The ESS measures daytime sleepiness; the SWIFT measures both sleepiness and fatigue.
- Comfort as measured by the S3NIV questionnaire and the previously piloted interface questionnaire at baseline, 3 months and 6 months. The S3NIV questionnaire measures respiratory symptoms, sleep quality and PAP therapy-related side effects.

#### Intervention device

The device will be a 3D-printed customised oronasal mask designed to be used with PAP therapy. The mask will be created using a 3D scanner, which captures a digital impression of the patient’s face; CAD will be used to create a mould, which will then be 3D printed, with the mould then used to make the mask. The part of the mask in contact with the participants’ skin will be silicon-based.

#### Comparator device

Conventional PAP masks will be used in the usual care group. An expert clinician will make a clinical assessment of the fit and decide on the conventional mask. They will all be full face masks. Depending on procurement and supply chain, a range of full-face masks will be utilised.

The PAP devices used will be CE/UKCA marked devices. A range of devices will be used depending on procurement and supply chain. Where feasible, the same manufacturer will be used throughout the study to ensure consistency in the data collected from the PAP devices.

The devices used in the manufacturing process will include the 3D scanner, Revopoint POP 2 (Revopoint, Hong Kong). A range of 3D printers will be used to ensure the sustainability of the research. This includes stereolithography printers (Form 3, FormLabs, Germany), selective laser sintering printers (Lisa Pro, Sinterit, Poland and Fuse 30+, FormLabs, Germany) and Polyjet printers (Object 260, Stratasys).

#### Intervention

Table 1 displays the schedule of assessment and interventions by visit. Participant randomisation will be undertaken centrally by the coordinating trial team site using Sealed Envelope: https://www.sealedenvelope.com

**Table 1:** Schedule of assessments and interventions by visit

Visit number	Conventional (control) mask group	Customised (intervention) mask group
1	Informed consent Facial scanning Physical assessment, including the presence of dermatitis, pressure ulcers, and eye symptoms. Vital measurements: SpO_2,_ weight, height, BMI. Demographics, Waterlow score, Co-morbidities, Rockwood frailty score Questionnaires: ESS, SWIFT and S3NIV PAP therapy device issued with conventional mask	Informed consent Facial scanning for production of customised 3D printed mask Physical assessment, including the presence of dermatitis, pressure ulcers and eye symptoms. Vital measurements: SpO_2,_ weight, height, BMI. Demographics, Waterlow score, Co-morbidities, Rockwood frailty score Questionnaires: ESS, SWIFT and S3NIV
1a		PAP therapy device issued with a customised mask
2	*Three months after the first visit* Physical assessment as per visit 1 Download data from the PAP machine *Questionnaires as per visit 1 plus interface questionnaire* *2-night cardiorespiratory sleep study* Adverse events review	*Three months after visit 1a* *Physical assessment as per visit 1* *Download data from the PAP machine* *Questionnaires as per visit 1 plus interface questionnaire* *2-night cardiorespiratory sleep study* *Adverse events review*
3	*Six months after visit 1* Physical assessment as per visit 1 Download data from the PAP machine *Questionnaires as per visit 2* *2-night cardiorespiratory sleep study* Adverse events review	*Six months after visit 1a* Physical assessment as per visit 1 Download data from the PAP machine *Questionnaires as per visit 2* *2-night cardiorespiratory sleep study* Adverse events review Issue conventional mask for ongoing treatment

BMIbody mass indexESSEpworth Sleepiness ScorePAPpositive airway pressureS3NIVS3 Non-Invasive VentilationSWIFTSleepiness-Wakefulness Inability and Fatigue Test

A 3D scan using a hand-held scanner of all participants’ faces will be collected for participants in both groups.

Those in the control arm will be fitted with a conventional PAP therapy mask and undergo an initial 10 min trial of PAP therapy to assess for mask leak, skin reactions and comfort scores. Once an acceptable mask is fitted, patients will go on to have PAP therapy as per their regular PAP regime. An additional visit (visit 1a) will be required for those in the intervention arm once their customised mask has been manufactured. Participants will undergo an initial 10 min trial of PAP therapy with the customised mask to assess for mask leak, skin reactions and comfort scores. If there are no serious adverse events (SAEs)/reactions during the PAP trial with the 3D-printed mask, patients will go on to have PAP therapy as per their normal PAP regime.

### Ethics and dissemination

#### Ethics

This protocol has been reviewed by the Hampshire B Research Ethics Committee (REC reference: 22/SC/0405), who have granted a favourable ethical opinion.

#### Consent

Participants who are unable to provide informed consent are excluded from the study. The research team will obtain consent before the study baseline measurements and after the participant’s information sheet review. Potential participants will be given a minimum of 24 hours to consider if they wish to participate in the study. Consent will be obtained from participants who are non-English-speaking using a translator.

#### Confidentiality

All data will be handled in accordance with the General Data Protection Regulation V.2018. Data collected will be password protected and only accessible by the research team.

Facial scans will be transferred securely from RFL to University College London (UCL) utilising nhs.net secure email. All members of the investigatory team who will need to access the facial scans of the participants will be provided with a nhs.net email account. Facial scans will be kept securely on UCL computers, password protected, and only accessible to the investigation team. Copies of facial scans will not be stored on UCL computers after the duration of the study and will be archived in accordance with UCL policy. Participants will give consent for the transfer of the facial scans and for their facial scans to be stored on UCL and RFL computer systems during the trial. Facial scans will not be used in any publications or other dissemination without participants’ prior explicit consent. The RFL Caldecott Guardian approved this protocol.

#### Sponsor

University College London Joint Research Office will act as the sponsor.

#### Audits and inspection

Trial-related monitoring, audits, Research Ethics Committee review and regulatory inspections will be conducted in line with UK policy framework for health and social care research.^25^

#### Trial closure

The end of the trial will be on completion of 160 participants consented and completion of the primary end point.

The trial will be suspended for investigations in the following circumstances:

**•** If a SAE is noted in 20% or more of the participants

#### Trial management

A trial management group will monitor all aspects of the conduct and progress of the trial, ensure the protocol is adhered to and take appropriate action to safeguard participants in the quality of the trial. The trial steering committee (TSC) will provide overall supervision of the trial and ensure it is conducted in accordance with the principles of good clinical practice and the relevant regulations. The TSC will meet 6 monthly during the time of the trial. A Data Management and Ethics Committee will meet annually to provide oversight on the need for any interim analysis and ensure participants’ safety by reviewing recruitment, adverse events and trial completion rates.

#### Publication policy

The results of this clinical investigation will be submitted for publication as an abstract at appropriate conferences and as a journal article to appropriate journals. Authorship will be determined in concordance with the International Committee of Medical Journal Editors authorship guidelines and UCL publication policy.

#### Timelines

The trial start date was January 2023, with an end date of May 2025 and anticipated publication date of December 2025.

## Discussion

The need for research focusing on developing patient-specific interfaces for PAP therapy that are acceptable to patients and avoid side effects such as leaks, pressure ulcers and ineffective PAP therapy has been recognised as a priority.^26^ The outcomes of this study will inform the sleep and ventilation community on the prospects of customised interfaces for PAP therapy. The outcomes from this trial could inform the design and manufacturing processes of future customised PAP therapy interfaces and support future trial conduct. Secondary outcomes and the assessment and diagnostic data from this trial could prove helpful in informing the sleep and ventilation community on the ongoing care and management of patients with SDB.

There are limitations to this trial. It is only feasible to assess medium-term (6-month) outcomes within the scope of the secured funding. Furthermore, due to the scope of the funding, it is not possible to blind assessors. The interventional nature of the trial design means it is not possible to blind participants. This is a single-site study, which is a further limitation potentially reducing the generalisability of the results. It has not been possible to include a health economics assessment due to the constraints of the secured funding. PPI groups have expressed frustration at the limitation of providing customised masks within a clinical trial and that due to regulations, the customised masks cannot be utilised outside of the clinical trial. It was not feasible or necessary (as agreed by the sponsor and the research site) for this clinical trial to be registered with the Medicine and Health Care Products Regulatory Agency. Thus, the results of this research cannot be used towards regulatory approval.
