# Supplementary material for: Clinical impact of customised positive airway pressure (PAP) therapy interfaces versus usual care in the treatment of patients with sleep-disordered breathing (3DPiPPIn): a randomised controlled trial protocol
Source: BMJ Open. 2024 Nov 14;14(11):e087234. doi: 10.1136/bmjopen-2024-087234 (PMC11575285; doi:10.1136/bmjopen-2024-087234)
Supplement: Supplementary file 1 [file bmjopen-2024-087234.reviewer_comments.pdf]

PEER REVIEW HISTORY

BMJ Open publishes all reviews undertaken for accepted manuscripts. Reviewers are asked to complete a checklist review form and are provided with free text boxes to elaborate on their assessment. These free text comments are reproduced below.

ARTICLE DETAILS

Title (Provisional)

The clinical impact of customised Positive Airway Pressure (PAP) therapy interfaces vs usual care in the treatment of patients with sleep disordered breathing (3DPiPPIn): a randomised controlled trial protocol

Authors

Mansell, Stephanie K.; Mandal , Swapna; Ridout, Deborah; Olsen, Oliver; GOWING, FRANCESCA; Kilbride, Cherry; Hilton, Stephen; Main, Eleanor; Schievano, Silvia

---

VERSION 1 - REVIEW

**Reviewer**	**1**
**Name**	**Yi, Hongliang**
**Affiliation**	**Shanghai 6th Peoples Hospital Affiliated to Shanghai Jiaotong University School of Medicine, Department of Otolaryngology Head and Neck Surgery & Center of Sleep Medicine**
**Date**	**06-May-2024**
**COI**	**no**

1.The protocol is a randomised control trial to assess the clinical impact of customised vs conventional interfaces in the treatment of patients with sleep disordered breathing, which is minimised by age >65 and ethnicity. Compared with previous studies, this protocol will add the sample size, and the stratification analysis of race and age stratification, may be afford more reliable evidence for CPAP with customised interfaces.

2.While it is important to reduce AHI with CPAP for OSA, comfort and compliance are equally important. As long as patients are willing to accept CPAP and undergo proper titration, AHI can generally be reduced to 5 or less. However, whether the patient is comfortable and can continue to use the CPAP device is the focus of our attention. Therefore, the residual AHI as the primary objective in this study is questionable.

3.The lack of blinding in this study may affect the judgment of the results.

4.Please provide a detailed calculation method for the number of samples

**Reviewer**	**2**
**Name**	**Elphick, Heather**
**Affiliation**	**The University of Sheffield**
**Date**	**29-May-2024**
**COI**	**No interests to declare**

The protocol is clearly described and the clinical need is well articulated. I have the following comments regarding the rationale behind the methodology chosen and some points for clarification.

1. Stratification of randomisation – the rationale for choosing ethnicity and age over and under 65 is not clear. Please could you expand on this and explain why other factors were not used in the stratification, for example underlying diagnosis, duration of usage in a 24-hour period?

2. Duration of usage in a 24-hour period is important to report even if not used for the stratification.

3. Devices – it is stated that all comparator devices will be full face-masks. This is not stated for the custom-made devices – if this is the case, it should be added and explained for clarity for the non-expert, ie is this an oronasal mask or a mask covering the whole face?

4. PPIE – this is well explained but no mention of the development of the custom-made masks – was PPIE involved in this?

5. Is there a reference for the previously piloted interface questionnaire and the EUPAP score?

6. Why is the control group scanned and what will happen to these scans?

7. Study limitations are clearly stated however, the fact that the study is taking place at a single centre could be seen as another limitation and should be added.

VERSION 1 - AUTHOR RESPONSE

Reviewer: 1

Dr. Hongliang Yi, Shanghai 6th Peoples Hospital Affiliated to Shanghai Jiaotong University School of Medicine

Comments to the Author:

1.The protocol is a randomised control trial to assess the clinical impact of

customised vs conventional interfaces in the treatment of patients with sleep disordered breathing, which is minimised by age >65 and ethnicity. Compared with previous studies, this protocol will add the sample size, and the stratification analysis of race and age stratification, may be afford more reliable evidence for CPAP with customised interfaces.

Thank you for taking the time to review our manuscript.

2.While it is important to reduce AHI with CPAP for OSA, comfort and compliance are equally important. As long as patients are willing to accept CPAP and undergo proper titration, AHI can generally be reduced to 5 or less. However, whether the patient is comfortable and can continue to use the CPAP device is the focus of our attention. Therefore, the residual AHI as the primary objective in this study is questionable.

We agree that comfort and concordance are important factors for patients receiving PAP therapy. This study underwent significant peer review during the securing of funding from the National Institute for Health Research (NIHR). Residual AHI was chosen as the primary outcome measure as, where interface fit is poor, residual AHI has been shown to be high. Furthermore, it is a measure of treatment effectiveness which can be used for both CPAP and NIV. The pros and cons of each potential primary outcome measure were discussed with PPI workshop attendees who agreed AHI was a suitable and acceptable primary outcome. We have added this justification to the “analysis” section of the manuscript. Comfort and concordance have been included as secondary outcome measures. We hope this addresses your concerns.

3.The lack of blinding in this study may affect the judgment of the results.

Thank you for your feedback, we have added this to the limitations.

4.Please provide a detailed calculation method for the number of samples

Details of the sample size are provided under the “Data management” heading and “sample size” subheading at lines 8-14 on page 9. Our statistician has reviewed this section of the manuscript following your comment, and this is already a detailed enough power calculation for replicability.

Reviewer: 2

Dr. Heather Elphick, The University of Sheffield
Comments to the Author:

The protocol is clearly described and the clinical need is well articulated. I have the following comments regarding the rationale behind the methodology chosen and some points for clarification.

1. Stratification of randomisation – the rationale for choosing ethnicity and age over and under 65 is not clear. Please could you expand on this and explain why other factors were not used in the stratification, for example underlying diagnosis, duration of usage in a 24-hour period?

Thank you for your helpful comments. We agree multiple stratification factors could have been chosen. On the advice of statisticians, this was limited to two in order to maintain a realistic and achievable sample size. Further stratifications would have increased the sample size and made the trial untenable. Based upon clinical expertise and published research, ethnicity and age were felt to be the biggest factors that would affect the facial geometry and thus have the biggest impact on the intervention(1, 2). We have added this justification to the "study design" section. In relation to the specific examples you have given, duration of usage in a 24-hour period was not used as participants are new to therapy. The linear regression will adjust for baseline AHI and thus underlying diagnosis is taken into account through the analysis. In order to keep the manuscript concise, we have not made an addition that justifies all the possible stratification we could have included but hope that the justification of the chosen stratification addresses your concerns adequately, but we are happy to add this if it is felt necessary.

2. Duration of usage in a 24-hour period is important to report even if not used for the stratification.

Thank you for your comment, this is already a secondary outcome measure. We refer to it as "Compliance with PAP therapy, measured as hours and percentage of days PAP therapy used >4 hours/night over 28 days" in the "assessment" section.

3. Devices – it is stated that all comparator devices will be full face-masks. This is not stated for the custom-made devices – if this is the case, it should be added and explained for clarity for the non-expert, ie is this an oronasal mask or a mask covering the whole face?

Thank you for your feedback. Both the customised and comparator masks are oronasal masks; we have amended this in the intervention section for clarity.

4. PPIE – this is well explained but no mention of the development of the custom-made masks – was PPIE involved in this?

Our interface questionnaire is collecting data specifically on product design, which will further guide development.

5. Is there a reference for the previously piloted interface questionnaire and the EUPAP score?

Thank you for your insights. There is no reference for the self-developed interface questionnaire. We have added a reference for the EUPAP.

6. Why is the control group scanned and what will happen to these scans?

The control group are scanned to reduce bias. These scans are stored as part of the trial.

7. Study limitations are clearly stated however, the fact that the study is taking place at a single centre could be seen as another limitation and should be added.

Thank you for your comments this has been added.

1. Farkas LG, Eiben OG, Sivkov S, et al. Anthropometric measurements of the facial framework in adulthood: age-related changes in eight age categories in 600 healthy white North Americans of European ancestry from 16 to 90 years of age. *J Craniofac Surg* 2004;15(2):288-98. doi: 10.1097/00001665-200403000-00027

2. Farkas LG, Katic MJ, Forrest CR, et al. International anthropometric study of facial morphology in various ethnic groups/races. *J Craniofac Surg* 2005;16(4):615-46. doi: 10.1097/01.scs.0000171847.58031.9e

---

VERSION 2 - REVIEW

**Reviewer**	**2**
**Name**	**Elphick, Heather**

Affiliation **The University of Sheffield**

Date **02-Oct-2024**

COI

Thankyou for clarification on the points raised. I'm happy that these have been resolved and recommend that the paper if accepted for publication.
